# Thrombospondin‐4 promotes bladder cancer cell migration and invasion via MMP2 production

**DOI:** 10.1111/jcmm.16463

**Published:** 2021-06-17

**Authors:** Kuang‐Yu Chou, An‐Chen Chang, Chao‐Yen Ho, Te‐Fu Tsai, Hung‐En Chen, Po‐Chun Chen, Thomas I‐Sheng Hwang

**Affiliations:** ^1^ Division of Urology Department of Surgery Shin‐Kong Wu Ho‐Su Memorial Hospital Taipei Taiwan; ^2^ Division of Urology School of Medicine Fu‐Jen Catholic University New Taipei Taiwan; ^3^ Translational Medicine Center Shin‐Kong Wu Ho‐Su Memorial Hospital Taipei Taiwan; ^4^ School of Medicine Institute of Traditional Medicine National Yang‐Ming University Taipei Taiwan; ^5^ Department of Biotechnology College of Health Science Asia University Taichung Taiwan; ^6^ Department of Medical Research China Medical University Hospital China Medical University Taichung Taiwan; ^7^ Department of Urology Taipei Medical University Taipei Taiwan

**Keywords:** bladder cancer, invasion, migration, MMP2, TSP4

## Abstract

Bladder cancer (BC) is the second most common urological tumour in Western countries. Approximately, 80% of patients with BC will present with non‐muscle invasive bladder cancer (NMIBC), whereas a quarter will have muscle invasive disease (MIBC) at the time of BC diagnosis. However, patients with NMIBC are at risk of BC recurrence or progression into MIBC, and an MIBC prognosis is determined by the presence of progression and metastasis. Matrix metalloproteinase 2 (MMP2), a type of matrix metalloproteinase (MMP), plays a major role in tumour invasion and is well‐characterized in BC prognosis. In BC, the mechanisms regulating MMP2 expression, and, in turn, promote cancer invasion, have hardly been explored. Thrombospondin‐4 (THBS4/TSP4) is a matricellular glycoprotein that regulates multiple biological functions, including proliferation, angiogenesis, cell adhesion and extracellular matrix modelling. Based on the results of a meta‐analysis in the Gene Expression Profiling Interactive Analysis 2 database, we observed that TSP4 expression levels were consistent with overall survival (OS) rate and BC progression, with the highest expression levels observed in the advanced stages of BC and associated with poor OS rate. In our pilot experiments, incubation with recombinant TSP4 promoted the migration and invasion in BC cells. Furthermore, MMP2 expression levels increased after recombinant TSP4 incubation. TSP4‐induced‐MMP2 expression and cell motility were regulated via the AKT signalling pathway. Our findings facilitate further investigation into TSP4 silencing‐based therapeutic strategies for BC.

## 1 INTRODUCTION

Bladder cancer (BC) is the second most common malignancy of the urinary tract, and it affects more than 380 000 people and causes 150 000 tumour‐related deaths every year worldwide.^1^ Despite the improvement of therapeutic options against BC available, recurrence occurs and progresses to invasive disease, with high mortality. Although most patients are diagnosed with non‐muscle invasive BC (NMIBC), approximately 50% of these patients will experience recurrence, which progresses to muscle invasive BC (MIBC). However, our understanding of the underlying mechanisms of such progression remains poor. Hence, the identification of novel prognostic factors for BC outcomes is essential for early diagnosis and target therapy.

Thrombospondin‐4 (TSP4) is a member of the extracellular calcium‐binding protein family, which comprises five members (TSP1 to TSP5).^2^ The thrombospondin family members were initially characterised as angiostatic factors due to their effects on endothelial cells via the regulation of vascular endothelial growth factor production and the subsequent promotion of angiogenesis.^3^, ^4^ The thrombospondin family of proteins are matricellular proteins that participate in diverse biological functions, including cell adhesion, cell migration, proliferation, cytoskeleton organization and the coordination of cell‐to‐cell interactions.^5^, ^6^, ^7^ Recently, TSP4 up‐regulation has been reported in several types of cancers, including breast cancer,^8^ prostate cancer^9^ and hepatocellular carcinoma.^10^ In addition, several recent studies have demonstrated the key roles of the thrombospondin family in tumour progression, particularly in metastasis.^11^, ^12^, ^13^ Although TSP4 has the capacity to modulate the extracellular matrix (ECM) in the tumour microenvironment in various ways,^14^ its roles in the initiation and progression of tumour growth have not been adequately explored.

Matrix metalloproteinases (MMPs) regulate ECM through the degradation of various ECM components, which, in turn, influence physiological functions including cell adhesion, proliferation, apoptosis, angiogenesis and epithelial‐to‐mesenchymal transition.^15^ By contrast, elevated MMP expression levels enhance several pathological processes, such as arthritis,^16^ cardiovascular disease,^17^ neurodegenerative disease^18^ and cancer.^15^ Most of the MMPs associated with BC have been discussed, including two gelatinases MMP2 and MMP9, which have been studied extensively considering their prognostic value.^19^ For example, MMP2 and MMP9 expression levels were significantly higher in tumour tissues than in normal tissue samples in BC.^20^ Although several studies have reported the prognostic value of MMP2 and MMP9, other researchers have reported inconsistent results,^21^ which attributes to these studies’ differences in the patients enrolled, detection methodologies adopted, and statistical methods adopted. Despite the inconsistent findings in clinical studies, the pro‐invasive functions of MMPs in BC progression have been validated.^22^

In the present study, we reveal that TSP4 is a candidate prognostic marker in BC. We noted that high TSP4 expression levels were correlated with poor prognosis in patients with BC. In addition, high TSP4 expression levels were associated with advanced tumour grades and poor survival rates in patients with BC In vitro studies have further demonstrated that the migratory potential of BC cells is significantly enhanced in response to TSP4 treatment, caused by MMP2 expression. A more detailed analysis revealed that TSP4 induces MMP2 protein expression via the AKT signalling pathway. Overall, targeting TSP4 can benefit BC treatment efforts.

## 2 MATERIALS AND METHODS

### 2.1 Cell culture

Human BC cell lines 5637 and T24 were obtained from the Bioresource Collection and Research Center (BCRC). Both cell lines were cultured in RPMI‐1640 medium supplemented with 10% foetal bovine serum, 2 mmol/L GlutaMAX‐1, 100 units/mL penicillin and 100 μg/mL streptomycin before being maintained at 37℃ under 5% CO_2_.

### 2.2 Datasets

The correlations among TSP4 expression, clinical stages, primary tumour status, tumour grades and overall survival were analysed for 412 tumour samples through the Gene Expression Profiling Interactive Analysis 2 (GEPIA2) (http://gepia2.cancer‐pku.cn/#index), Gene Expression database of Normal and Tumor tissues (GENT2) (http://gent2.appex.kr/gent2/) tool.

### 2.3 Cell viability

Cells (3 × 10^4^/well) were seeded into 48‐well plates with 200 μL of culture medium. Resazurin‐based cell viability assay was assessed after TSP4 or MMP2 siRNA treatment for 24 hours. In brief, the BC cells were incubated with 20 μL of resazurin solution for 4 hours at 37℃. The fluorescent signal was measured using a 550 nm excitation filter and a 600 nm emission filter by VARIOSKAN LUX multimode microplate reader (Thermo Fisher Scientific).

### 2.4 Transfection of MMP2 siRNA

Cells (3 × 10^5^/well) were seeded into 6‐well plates. Viromer RED (Lipocalyx), a plasmid transfection reagent, was used to transfect MMP2 siRNA in cells for 24 hours, followed by TSP4 (R&D Systems) treatment for an additional 24 hours. Cell motility in the cell samples was evaluated using a Transwell migration assay.

### 2.5 Transwell migration and invasion assay

Cell migration and invasion assays were performed using Transwell inserts (8‐μm pore size; Costar) in 24‐well plates. The invasion assay was pre‐coated with 30 μL of Corning Matrigel matrix (Corning) in the upper chamber for 30 minutes. Cells (1 × 10^4^ in 200 μL of serum‐free medium) were seeded into the upper chamber, while 300 μL of serum‐free medium was placed in the lower chamber. After 24 hours, the migrated and invaded cells were stained with 0.05% crystal violet and photographed.

### 2.6 Wound healing assay

Cells (1 × 10^5^ cells/well) were seeded onto 12‐well plates. After 24 hours, the confluent monolayer of culture was scratched with a fine pipette tip, followed by TSP4 treatment for 24 hours. The migrated cells were visualised under microscopy and quantified by counting cell numbers.

### 2.7 Western blot analysis

The protein samples were prepared from cell lysates and resolved by SDS‐PAGE. Subsequently, protein samples were transferred to Immobilon polyvinyldifluoride membranes and blocked with 4% bovine serum albumin for 1 hour at room temperature. The blots were probed with rabbit anti‐human antibodies against candidate signal pathway proteins—pAKT (1:1000; GeneTex), AKT (1:1000; GeneTex), MMP2 (1:1000; GeneTex), and MMP9 (1:1000; Abcam)—for 2 hours at room temperature. After three washes with tris‐buffered saline containing Tween 20, the blots were subsequently incubated with a donkey anti‐rabbit peroxidase‐conjugated secondary antibody (1:3000; Cell Signaling Technology) for 1 hour at room temperature. Protein levels on the blots were detected with enhanced chemiluminescence using Kodak X‐OMAT LS film (Eastman Kodak) and quantified using a computing densitometer and ImageQuant (Molecular Dynamics).

### 2.8 Quantitative real time polymerase chain reaction

Total RNA samples were isolated from BC cells and converted to cDNA. Quantitative RT‐ PCR analyses were performed using Taqman one‐step PCR Master Mix (Applied Biosystems). We added 100 ng of total cDNA per 25‐µL reaction with sequence‐specific primers (MMP2, MMP9, and β‐actin). qRT–PCR assays were conducted in triplicate on a StepOnePlus sequence detection system (Thermo Fisher Scientific). The cycling conditions were conducted under identical conditions as those in a previous study.^23^ The threshold was set above the non‐template control background and within the linear phase of target gene amplification to calculate the cycle number at which the transcript is detected (denoted C_T_).

### 2.9 Immunohistochemistry

The human BC tissue arrays (T124b and BL2081a) were obtained from US Biomax and then deparaffinized in xylene, rehydrated in a graded alcohol series, and washed in deionized water. After antigen retrieval, the intrinsic peroxidase activity and non‐specific antibody binding sites were blocked by 3% hydrogen peroxide and 3% bovine serum albumin (BSA), respectively. Sections were incubated with a specific primary antibody (TSP4; 1:200; Sigma‐Aldrich) for 2 hours. After undergoing washing with PBST, the secondary antibody was applied for 1 hour at room temperature. Stained sections were detected with 3,3′‐diaminobenzidine tetrahydrochloride (DAB), then counterstained with haematoxylin and eosin and observed under a light microscope. IHC tissues were grouped into categories of negative, weak, moderate and strong staining.

### 2.10 Statistics

All experiments were performed at least three times, each time in triplicate. A statistical comparison between two samples was performed using the Student's *t* test. One‐ and two‐way ANOVA followed by Bonferroni's post hoc comparison tests were used to compare the means of more than two groups. The results are presented in terms of the mean ± standard deviation. Statistical significance was indicated if *P* < .05.

## 3 RESULTS

### 3.1 TSP4 is positively correlated with cancer progression in BC

**FIGURE 1:**
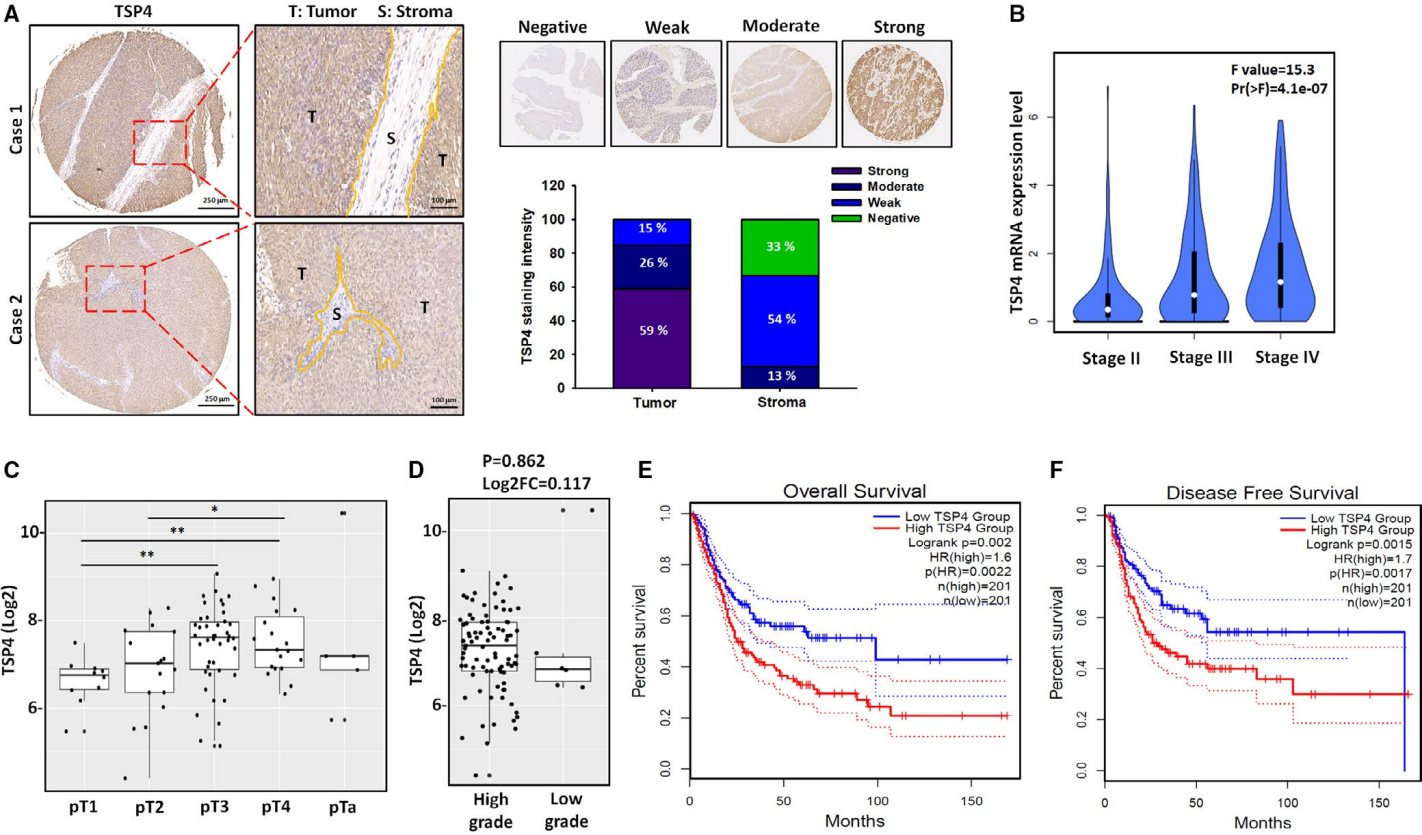
TSP4 expression in human BC tissues. A, Representative images of immunohistochemical staining for Thrombospondins‐4 (TSP4) in human bladder cancer (BC) tissues. B, The relationship between TSP4 expression and clinical stages of BC was analysed using the Gene Expression Profiling Interactive Analysis 2 (GEPIA2) web server. The F value is representative of the *F* test. C and D, The Gene Expression database of Normal and Tumor tissues 2 (GENT2) web server was used to analyse the correlations among TSP4 expression, primary tumour status, and tumour grade. E and F, The overall survival and disease‐free survival rates were analysed between high and low TSP4 groups in the GEPIA2 web server. All data are expressed in terms of mean ± SD in triplicate samples. *P *< *.05, **P < .01 relative to the control group

TSP4, a secreted ECM protein, is associated with tissue remodelling, inflammation and angiogenesis activities.^24^ However, its role in BC tumour progression is poorly understood. Our histopathological examination of TSP4 protein expression revealed higher levels in the tumour tissues of patients with BC than in stroma regions (Figure 1A), which indicated that TSP4 was specifically expressed in BC tissues. We further explored the clinical importance of TSP4 in human BC. Our analysis of human bladder urothelial carcinoma datasets using the online Gene Expression Profiling Interactive Analysis 2 (GEPIA2) and Gene Expression database of Normal and Tumor tissues 2 (GENT2) tool revealed that TSP4 was highly associated with clinical disease stage (^****^ *P* < .0001) (Figure 1B) and primary tumour status (T) (Figure 1C; Table. 1), respectively. TSP4 expression was slightly elevated in high‐grade BC tumours than in low‐grade BC tumours; however, the differences were not significant (*P* = .862) (Figure 1D). To assess the prognostic value of TSP4 expression in patients with BC, we investigated the associations among the TSP4 expression, overall survival (OS), and disease‐free survival (DFS). We observed higher levels of TSP4 expression in patients with BC, which was also correlated with poor OS (^**^ *P* = .002) (Figure 1E) and DFS (^**^ *P* = .0015) (Figure 1F). The results suggest that TSP4 is overexpressed in BC and is correlated with clinical stage and poor prognosis.

**TABLE 1:** Significant test results between TSP4 and clinical tumour status by two‐sample *t* test

Tissue	*P*‐value	Log2FC
pT1 vs pT2	.464	0.23
pT1 vs pT3	.002**	0.798
pT1 vs pT4	.001**	0.889
pT1 vs pTa	.340	0.865
pT2 vs pT3	.063	0.568
pT2 vs pT4	.042*	0.660
pT2 vs pTa	.479	0.635
pT3 vs pT4	.685	0.092
pT3 vs pTa	.937	0.067
pT4 vs pTa	.977	−0.024

*P < .05, **P < .01 relative to the pT1 or pT2 group.

### 3.2 TSP4 promotes cell migration and invasion in BC cells

**FIGURE 2:**
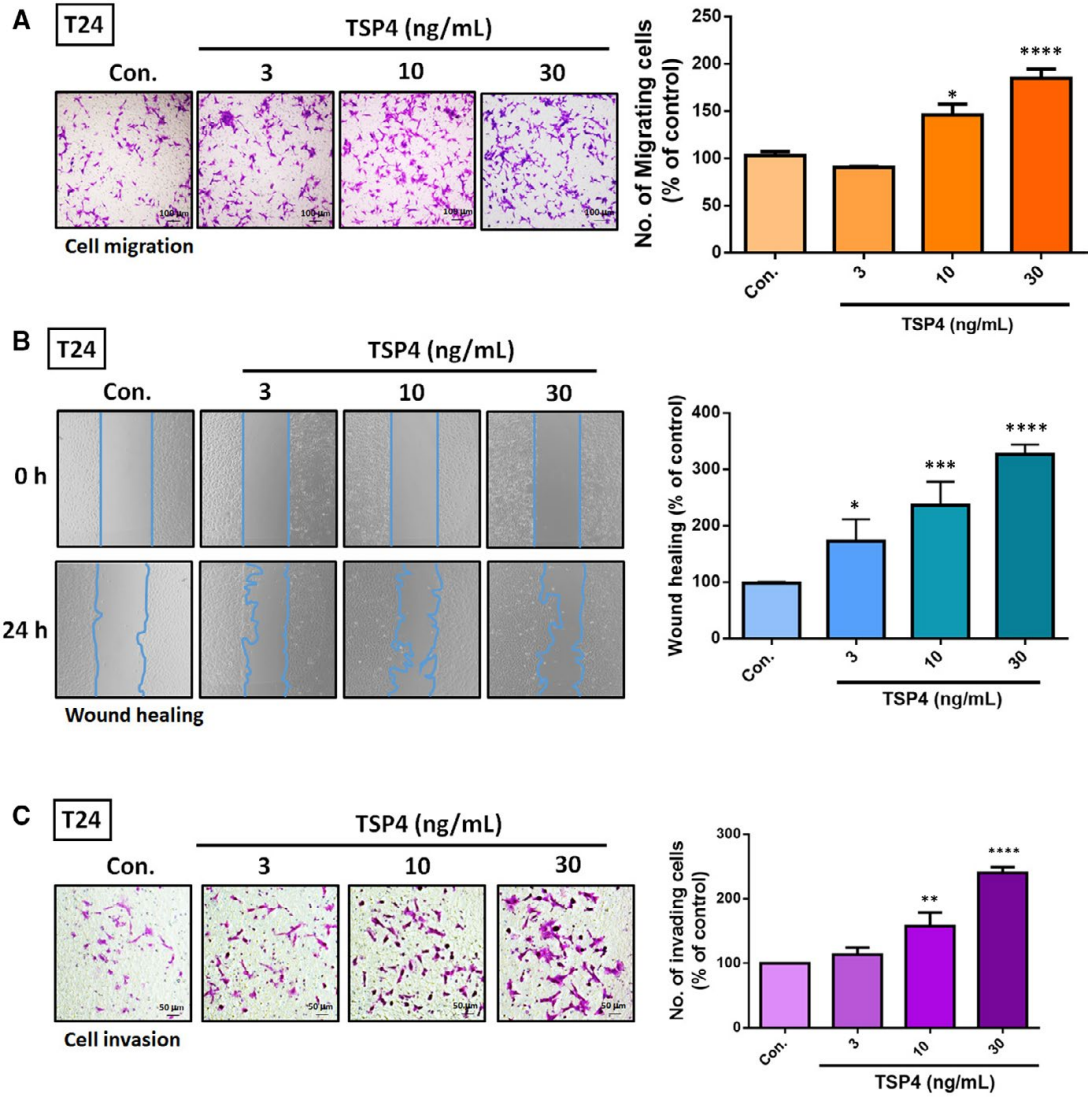
TSP4 induces cell migration and invasion in BC. A and B, Treatment of T24 cells with different concentrations of recombinant TSP4 protein (0‐30 ng/mL) for 24 h. The cell migratory capacity was evaluated using a Transwell assay and wound healing assay. C, Incubation of T24 cells with recombinant TSP4 protein (0‐30 ng/mL) for 24 h; cell invasion was evaluated using a Transwell assay. The cell numbers of migrating and invading cells were quantified. All data are expressed in terms of mean ± the SD of triplicate samples. *P *< *.05, **P < .01, ***P < .001, ****P < .0001 relative to the control group

Tumour progression involves sequential steps, such as the coordination of cell proliferation, adhesion, survival, migration and angiogenesis.^25^ In this study, we investigated the mechanism through which TSP4 regulates cell migration in BC. The Transwell migration assay and the wound healing assay revealed that TSP4 induced cell motility in the T24 BC cell line (Figure 2A,B). Furthermore, treatment of T24 cells with different concentrations of TSP4 enhanced cell invasiveness (Figure 2C). Overall, the results demonstrate that TSP4 has the capacity to regulate BC cell movement.

### 3.3 MMP2 is required for TSP4‐induced cell migration and invasion in BC cells

**FIGURE 3:**
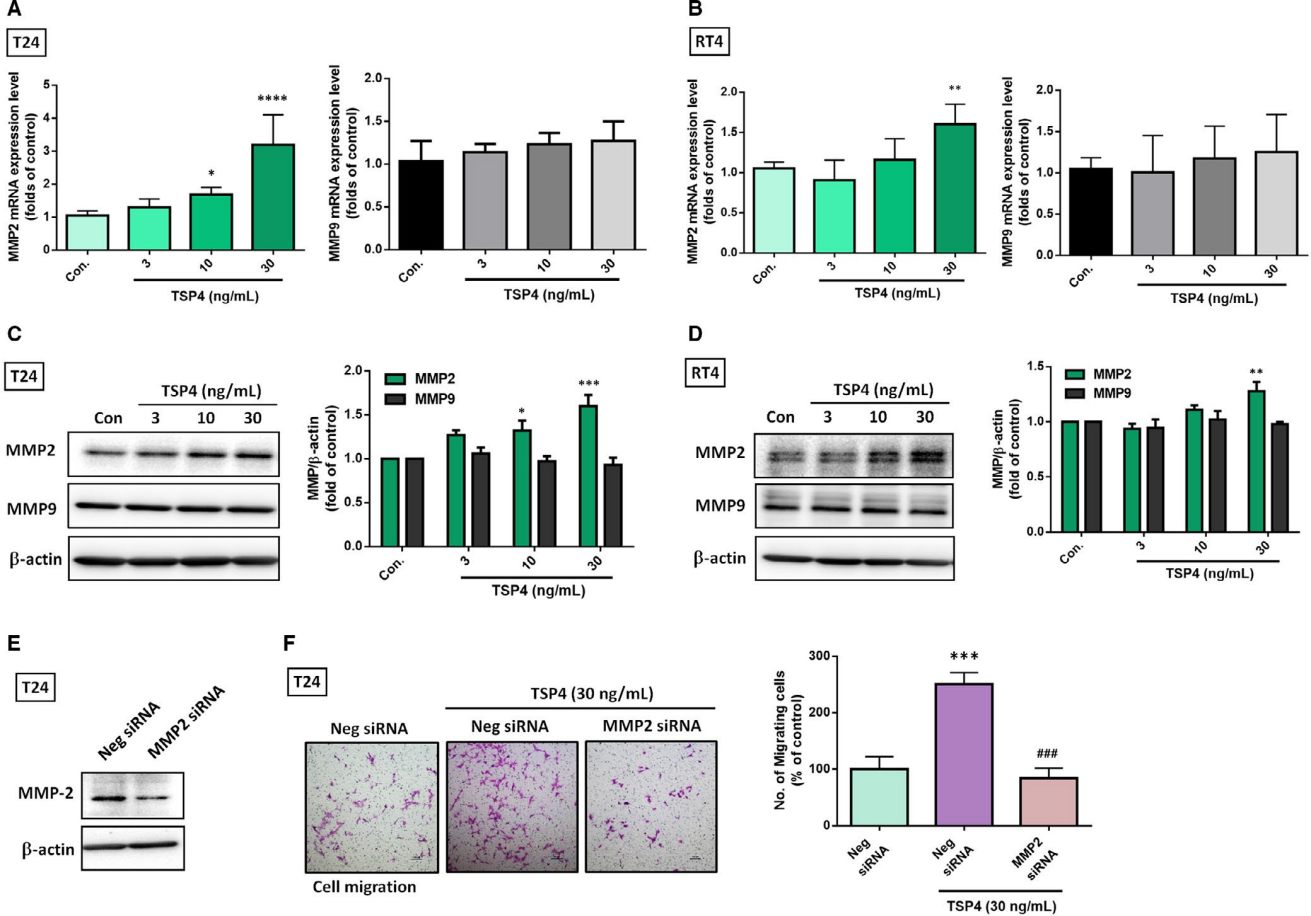
TSP4 promotes MMP2 expression in BC cells. A‐D, Treatment of T24 and RT4 cells with different concentrations of TSP4 protein (0‐30 ng/mL) for 24 h. The levels of TSP4 mRNA and protein expression were evaluated using western blot and qRT‐PCR assay, respectively. E, Transfection of T24 cells with negative (Neg) or MMP2 siRNA (10 μmol/L) for 24 h followed by with or without TSP4 for another 24 h. The cell migratory capacity was assessed using a Transwell assay and quantified by counting the migrated cells. All data are expressed in terms of the mean ± the SD of triplicate samples. *P *< *.05, **P < .01, ***P < .001, ****P < .0001 relative to the control or Neg siRNA group. ^###^ *P* *< *.001 relative to the TSP4‐treated group

Numerous studies have implicated MMP2 and MMP9 as active contributors of malignant progression because they enhance cancer cell migration and invasion.^22^ Therefore, we confirmed that MMP2 and MMP9 are critical mediators of TSP4‐regulated cell migration and invasion. Our treatment of BC cell lines T24 and RT4 with various concentrations of recombinant TSP4 protein revealed that TSP4 induced MMP2 protein and mRNA expression; however, no effect was seen on MMP9 expression (Figure 3A‐D). To investigate whether the increase in MMP2 influenced TSP4‐regulated cell motility, we transfected MMP2 siRNA in BC cells to knock down its expression (Figure 3E). According to the results, MMP2 siRNA inhibited cell motility without affecting cell viability (Figure S1A,B). Moreover, TSP4‐induced cell migration halted by MMP2 siRNA (Figure 3F). Therefore, MMP2, but not MMP9, is required for TSP4‐induced cell migration and invasion to occur in BC cells.

### 3.4 TSP4 and MMP2 expression is positively correlated with BC tumors

**FIGURE 4:**
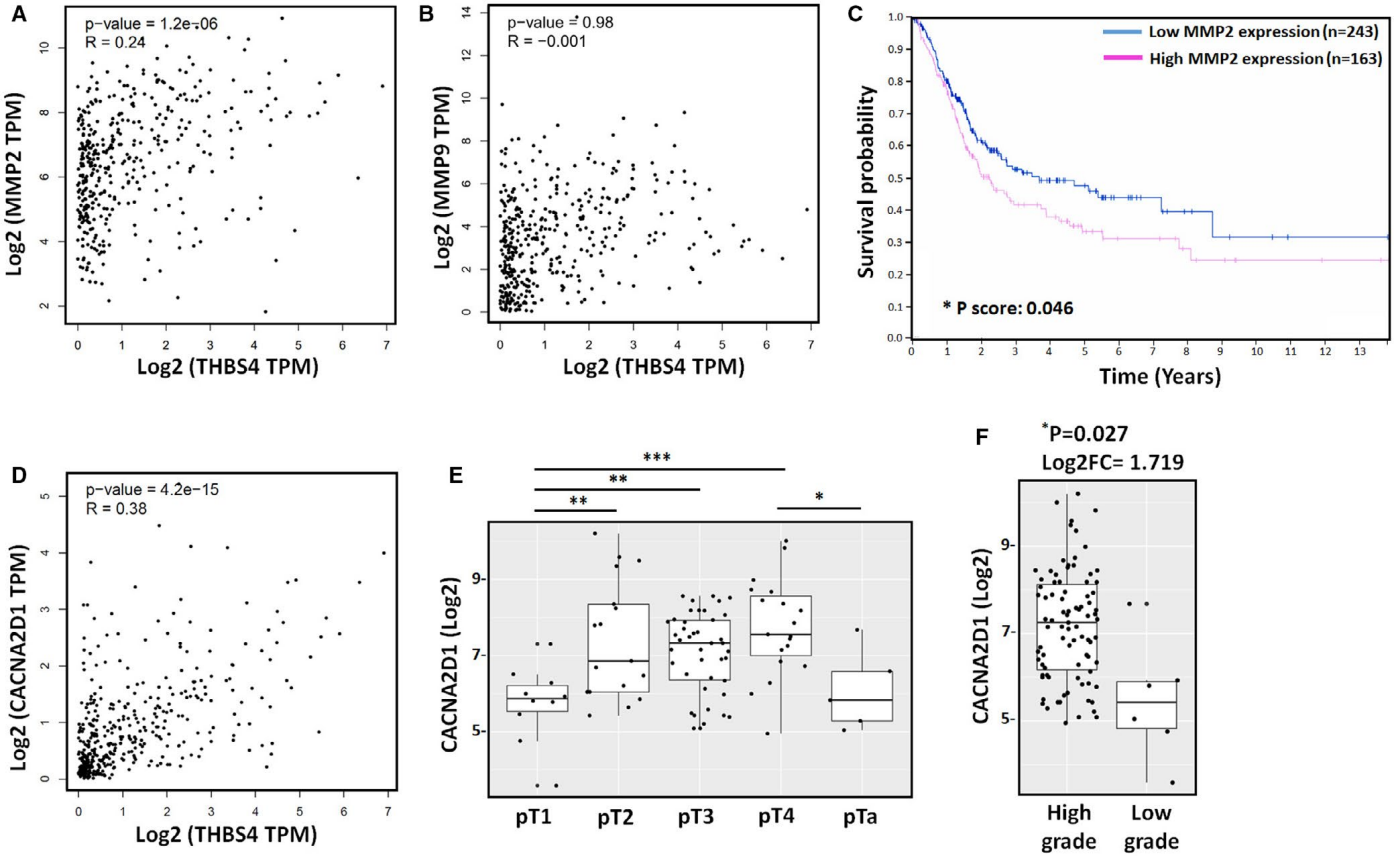
MMP2 expression positively associates with TSP4 and poor survival rate. A and B, Correlations among TSP4 (*THBS4*), MMP‐2, and MMP9 expression levels in BC samples analysed using the GEPIA2 web server. C, Overall survival rates in patients with BC with high and low MMP2 expression levels were analysed using the Human Protein Atlas web server. The 406 patients were divided based on the level of FPKM (number Fragments Per Kilobase of exon per Million reads) expression into high and low MMP2 expression groups. The cut‐off value was set to 68.82. D, The correlation between TSP4 and gabapentin receptor α2δ–1 (*CACNA2D1*) expression level in BC samples analysed using the GEPIA2 web server. E and F, The GENT2 web server was used to analyse the correlations among *CACNA2D1* gene expression, primary tumour status, and tumour grade. All data are expressed in terms of the mean ± the SD of triplicate samples. *P *< *.05, **P < .01, ***P < .001 relative to the control group

To further validate the TSP4, MMP2, and MMP9 associations in human BC tumours, we analysed the correlations among TSP4, MMP2 and MMP9 expression in human BC tumours by analysing The Cancer Genome Atlas (TCGA) database for urothelial bladder carcinoma datasets on the GEPIA2 web server. As expected, TSP4 correlated positively with MMP2 in BC tumours (^****^ *P* < .0001, *R* = .24) (Figure 4A); however, no correlations between TSP4 and MMP9 levels in BC tumours were noted (*P* = .98, *R* = −0.1) (Figure 4B). The results agree with previous observations of TSP4 promoting MMP2 expression but not MMP9 expression (Figure 3A‐C). Notably, we observed high MMP2 expression levels, which were significantly and negatively correlated with OS rate (Figure 4C).

The α2δ–1 subunit has been identified as TSP4‐specific receptor that contributes to regulating numerous cellular functions, such as endothelial cell proliferation and glial‐induced synapse formation.^26^, ^27^ In the present study, we analysed the clinical importance of α2δ–1 subunit in BC tumours. We observed that TSP4 and α2δ–1 had a positive correlation (^****^ *P* < .0001, *R* = .38) (Figure 4D). Moreover, α2δ–1 was highly associated with primary tumour status (Figure 4E; Table. 2) and high‐grade BC tumours (^*^ *P* = .027) (Figure 4F). Such findings indicate that (a) TSP4 expression is positively correlated with α2δ–1 and MMP2 expression in BC tumour tissues and (b) high MMP2 levels are associated with poor OS rate.

**TABLE 2:** The clinical correlation between CACNA2D1 expression and clinical tumour status

Tissue	*P*‐value	Log2FC
pT1 vs pT2	.002**	1.675
pT1 vs pT3	.002**	1.336
pT1 vs pT4	<.001***	1.985
pT1 vs pTa	.567	0.344
pT2 vs pT3	.421	−0.339
pT2 vs pT4	.522	0.31
pT2 vs pTa	.055	−1.331
pT3 vs pT4	.064	0.649
pT3 vs pTa	.107	−0.992
pT4 vs pTa	.021*	1.641

*P < .05, **P < .01, ***P < .001 relative to the pTa or pT1 group.

### 3.5 The AKT pathway is involved in the pro‐invasive function of TSP4 in BC

**FIGURE 5:**
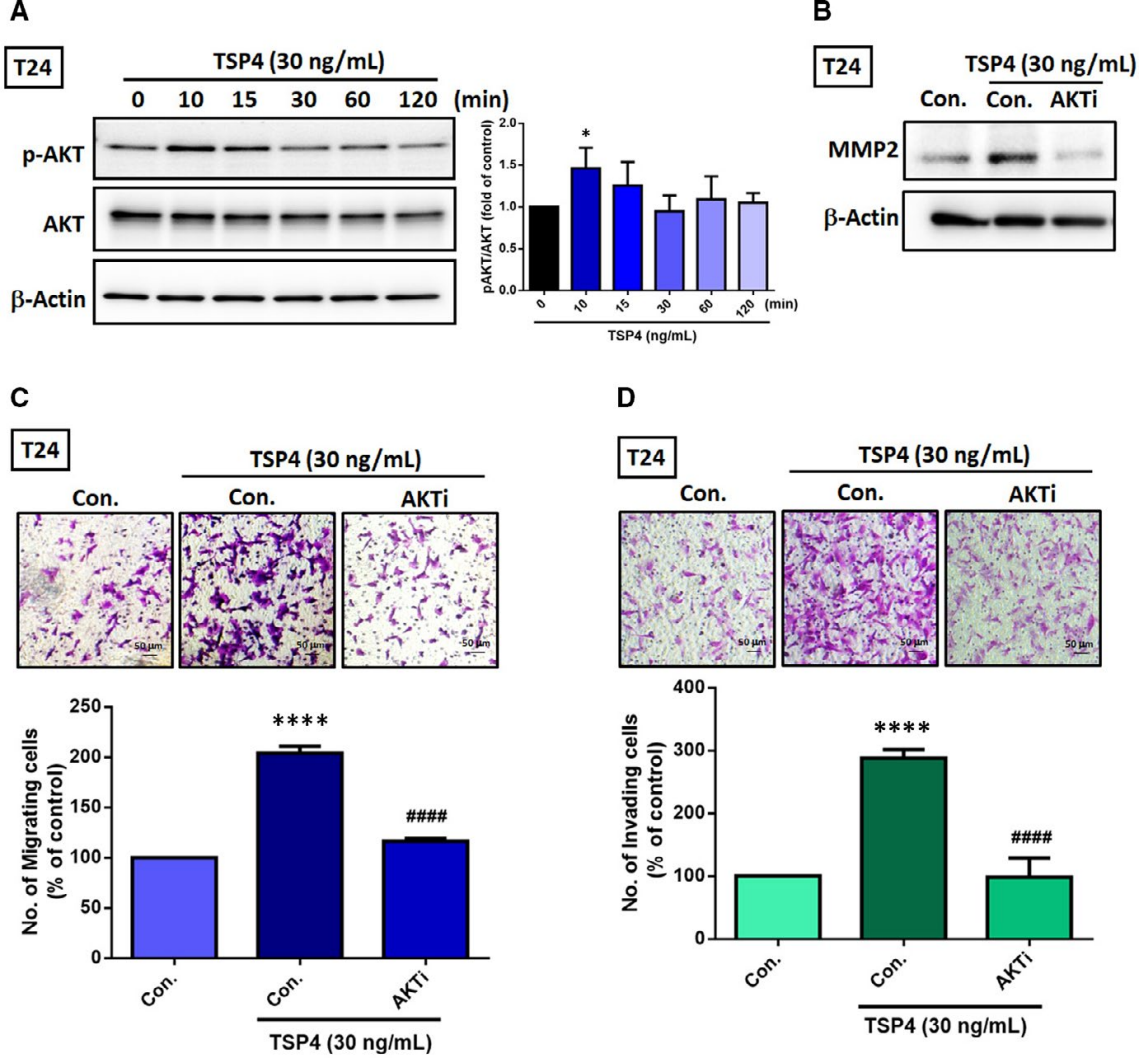
AKT pathway is essential in TSP4‐regulated pro‐invasive function. A, Incubation of T24 cells with recombinant TSP4 protein at the indicated time points, p‐AKT and total AKT protein expression were evaluated using a western blot assay. B, Pretreatment of T24 cells with AKTi (0.5 μmol/L) for 30 min followed by with or without TSP4 (30 ng/mL) for 24 h. The MMP2 protein levels were evaluated using a western blot assay. C and D, T24 cells were treated with AKTi (0.5 μmol/L) for 30 min, followed by TSP4 (30 ng/mL) for 24 h. Cell migration and invasion were measured using a Transwell assay. The cell numbers of migrating and invading cells were quantified. All data are expressed in terms of the mean ± the SD of triplicate samples. ****P *< *.0001 relative to the control group. ^####^ *P* *< *.0001 relative to the TSP4‐treated group

We investigated the underlying signalling pathways in the TSP4‐treated BC cells. AKT pathway activation is considered to play key roles in BC, such as promoting chemoresistance^28^ and regulating tumour growth.^29^ We detected high levels of AKT phosphorylation after 10 minutes of TSP4 incubation in BC cells. Subsequently, AKT phosphorylation levels were stabilised and restored to baseline levels within 30‐120 minutes (Figure 5A). T24 cells were then cotreated AKTi (0.5 μmol/L) and TSP4 for 24 hours and resazurin‐based cell viability assay was assessed. AKT functions as a critical regulator of cell survival and proliferation.^30^ Unsurprisingly, cell viability was slightly repressed by AKTi; however, the differences between cotreatment and TSP4 alone were not significant (Figure S1C). Furthermore, we observed that AKTi suppressed the TSP4‐induced increase in MMP2 protein levels (Figure 5B). In addition, AKTi halted TSP4‐regulated cell migration (Figure 5C) and invasion (Figure 5D). Based on these results, we conclude that TSP4’s induction of the invasive and migratory potential of BC is regulated via the AKT signalling transduction pathway.

## 4 DISCUSSION

A growing body of research has indicated that TSP4 regulates fibrosis and myocardium remodelling,^31^ is correlated with vascular inflammation and atherogenesis,^32^ controls the composition of the ECM surrounding muscles and tendons,^33^ and influences the risk of cardiovascular disease.^34^ In addition, TSP4 has attracted much recent interest and has been associated with various cancers. However, the mechanisms underlying such associations have remained unclear. Our results identify TSP4 as a novel mediator of cell migration and invasion in human BC.

TSPs represent a family of multidomain proteins that contain several types of repeated sequence modules, such as type 1, 2 and 3 repeats.^14^ Each domain has a specific function through interactions with ECM proteins, receptors and ligands—which subsequently activate signalling pathways that regulate cellular physiological functions or control tissue remodelling.^35^ Notably, TSP1 has been reported to participate in the regulation of MMP activity through direct TSP1/MMPs binding by the type 1 repeat domain^35^ as well as through indirect mechanisms, such as transcriptional and post‐translational regulation.^36^ However, TSP4 does not have a type 1 repeat domain, which lacks the capacity to bind with MMPs. Our results demonstrate that TSP4 is a novel upstream mediator of MMP2 through the AKT signalling pathway. Interestingly, overexpression of TSP4 in bone marrow stromal cells (BMSCs) enhanced angiogenesis by expanding the expression of the MMP2 and MMP9 proteins in cerebral ischemic penumbra of middle cerebral artery occlusion model. The TSP4‐BMSCs fusion provides an improved therapeutic effect on post‐stroke angiogenesis.^37^ The findings imply that TSPs‐regulated MMPs functions can be broadly characterised as having direct and indirect effects depending on the domains in the TSPs. In BC, numerous subtypes of MMPs containing MMP1, MMP7, MMP9 and MMP11 have investigated a positive correlation between increased levels of MMPs and tumour progression.^38^ Overexpression of MMP2 and MMP9 favour tumour recurrence in patients with stage T1 BC.^39^ Moreover, urinary MMP1 has been considered as a prognostic factor predicting advanced stage or grade and poor BC survival.^40^ In the present study, TSP4 up‐regulated MMP2 but not MMP9 expression, which in turn elevated cell migration and invasion in human BC. All these findings suggest that MMPs are attractive targets for BC treatment.

Integrins α_M_β_2_, α_V_β_3_ and gabapentin receptor α2δ–1 have been identified as TSP4‐specific receptors that participate in the regulation of cell adhesion, proliferation, migration and proinflammatory activities.^26^, ^41^ In addition, human neutrophils have been reported to adhere to the TSP4 variant (P387) via integrins α_M_β_2_, and partially via α_V_β_3_, and the receptors subsequently activate proinflammatory cytokine IL‐8 secretion and H_2_O_2_ production. These results suggest that the TSP4 variant (P387) creates a microenvironment that is rich in proatherogenic stimuli and conducive for lesion development.^41^ In addition, gabapentin receptor α2δ–1 facilitates endothelial cell proliferation in response to TSP4.^26^ The receptor α2δ–1 has also been recognized as a neuronal TSP receptor, and is required for TSP mediated glial‐induced synaptogenesis.^27^ These observations confirm that integrins and gabapentin receptor α2δ‐1 may play an important role in TSP4‐regulated cellular physiological functions. In this study, we found that receptor α2δ–1 level has a positive correlation with TSP4 expression, primary tumour status and tumour grade in human BC tumour tissues. However, we did not investigate whether the receptors are involved in TSP4‐mediated MMP2 expression and cell motility; such involvement requires further investigation.

Tumour angiogenesis is required to provide nutrients and oxygen to malignant tissues, and it plays a key role in tumour growth, maintenance, and metastasis.^42^ Therefore, developing inhibitors that target tumour vasculature is considered a promising therapeutic approach. The anti‐angiogenic properties of TSP1 and TSP2 have been reported based on numerous in vivo and in vitro assays.^43^ In addition, a TSP1‐derived peptide (d‐reverse amKRFKQDGGWSHWSPWSSac) impeded brain tumour growth through decreased blood vessel formation rate and anti‐proliferative effects against tumour cells.^44^ However, TSP4 has a contrasting function in angiogenesis regulation, in that it promotes angiogenesis in a cancer model. Furthermore, angiogenesis was decreased in *THBS4* ^−/−^ mice compared with the case in wild‐type mice.^26^ The potential reason for the contrasting functions is that TSP1 and TSP2 have similar structural domains containing type I repeats, which contribute to these anti‐angiogenic properties. By contrast, TSP4 lacks type I repeats, therefore, loses its anti‐angiogenic function. Our study presents novel findings demonstrating that TSP4 is a potential target for treating BC. Based on the analysis of TCGA database, we noted that TSP4 was positively associated with clinical stage, poor OS, and DFS rate in patients with BC. In addition, our in vitro experiments demonstrate TSP4 significantly induced cell motility and invasiveness in BC cells through AKT signalling pathway. Importantly, AKT signalling pathway is crucial for bladder cancer initiation, progression and chemoresistance.^28^, ^45^, ^46^ These results suggest that therapeutic targeting of the TSP4/AKT axis represents a novel and much‐needed approach for improving the outcome of patients with BC.

**FIGURE 6:**
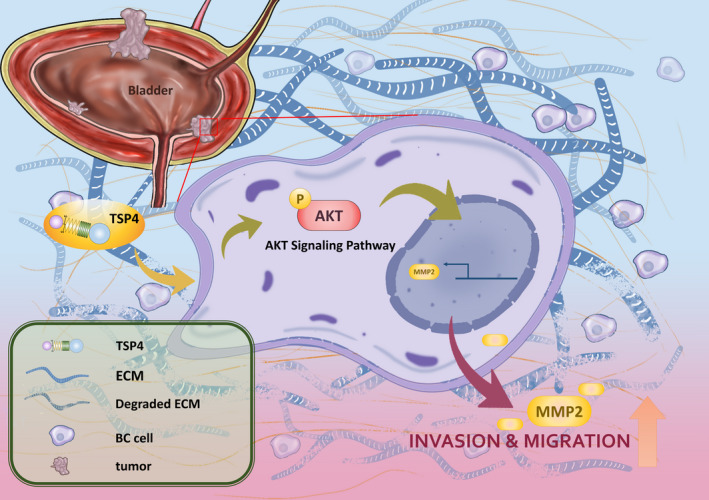
Schematic of how TSP4 regulates cell migration and invasion through MMP2 expression promotion

In conclusion, we illustrate the role of TSP4 in regulating cancer cell motility. We also identify the AKT signalling pathway to be involved in TSP4‐promoted MMP2 expression by way of enhancing cell migration and invasion activities (Figure 6).

## CONFLICT OF INTEREST

The authors of this article declare no conflicts of interest.

## AUTHOR CONTRIBUTION

**Kuang‐Yu Chou:** Conceptualization (equal); Data curation (equal); Investigation (equal); Writing‐original draft (equal); Writing‐review & editing (equal). **An‐Chen Chang:** Conceptualization (equal); Investigation (equal); Methodology (equal); Software (equal); Writing‐original draft (equal). **Chao‐Yen Ho:** Formal analysis (equal); Project administration (equal). **Te‐Fu Tsai:** Formal analysis (equal); Resources (equal). **Hung‐En Chen:** Project administration (equal). **PoChun Chen:** Data curation (equal); Software (equal); Supervision (equal). **Thomas I‐Sheng Hwang:** Supervision (equal); Writing‐review & editing (equal).

## Supporting information

Figure S1

## Data Availability

The data that support the findings of this study are available from the corresponding author upon reasonable request.
