# Supplementary material for: Thrombospondin‐4 promotes bladder cancer cell migration and invasion via MMP2 production
Source: J Cell Mol Med. 2021 Jun 17;25(13):6046–55. doi: 10.1111/jcmm.16463 (PMC8406484; doi:10.1111/jcmm.16463)
Supplement: Supplementary file 1 — Figure S1 [file JCMM-25-6046-s001.pdf]

Supplementary Figure 1

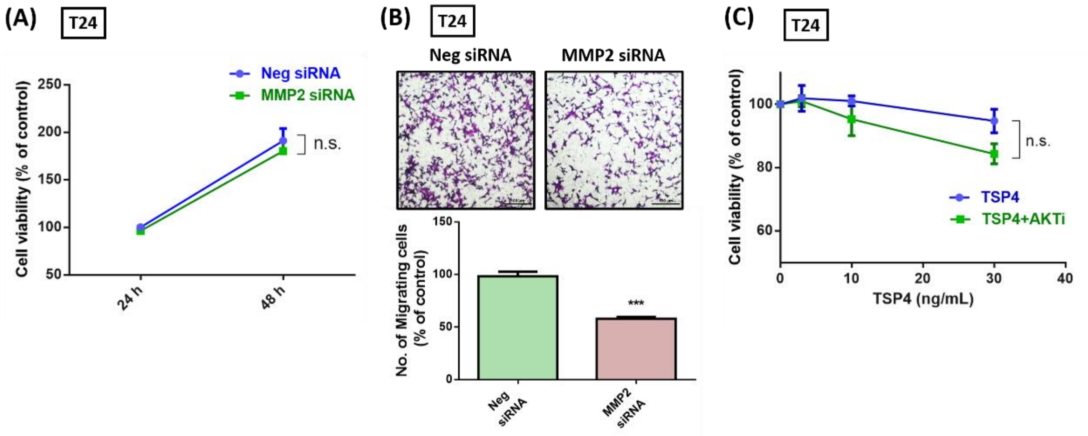

Fig. S1. The effect of MMP2 in regulating BC cell viability and migration.

(A&B) Transfection of T24 cells with control or MMP2 siRNA (10 μM) for 24 h. Cell viability was assessed using resazurin cell viability kit. Cell migration was measured by Transwell assay and quantified by counting migrated cells. (C) T24 cells were pretreated with or without AKTi (0.5 mM) for 30 min, followed by TSP4 (0-30 ng/mL) incubation for 24 h. Cell viability was assessed by resazurin cell viability kit. All data are expressed in terms of the mean ± the SD of triplicate samples. $^{***}P < 0.001$ relative to Neg siRNA group; n.s., not significant.
